# In vivo assessment of muscle mitochondrial function in healthy, young males in relation to parameters of aerobic fitness

**DOI:** 10.1007/s00421-019-04169-8

**Published:** 2019-06-08

**Authors:** Bart Lagerwaard, Jaap Keijer, Kevin K. McCully, Vincent C. J. de Boer, Arie G. Nieuwenhuizen

**Affiliations:** 1 Human and Animal Physiology, Wageningen University and Research, PO Box 338, 6700AH Wageningen, The Netherlands; 2 Department of Kinesiology, University of Georgia, Athens, USA

**Keywords:** Mitochondrial capacity, NIRS, EPOC, Oxidative metabolism, Muscle mitochondria

## Abstract

**Purpose:**

The recovery of muscle oxygen consumption (m$\dot{V}$O_2_) after exercise provides a measure of skeletal muscle mitochondrial capacity, as more and better-functioning mitochondria will be able to restore m$\dot{V}$O_2_ faster to the pre-exercise state. The aim was to measure muscle mitochondrial capacity using near-infrared spectroscopy (NIRS) within a healthy, normally active population and relate this to parameters of aerobic fitness, investigating the applicability and relevance of using NIRS to assess muscle mitochondrial capacity non-invasively.

**Methods:**

Mitochondrial capacity was analysed in the gastrocnemius and flexor digitorum superficialis (FDS) muscles of eight relatively high-aerobic fitness ($\dot{V}$O_2_peak ≥ 57 mL/kg/min) and eight relatively low-aerobic fitness male subjects ($\dot{V}$O_2_peak ≤ 47 mL/kg/min). Recovery of whole body $\dot{V}$O_2_, i.e. excess post-exercise oxygen consumption (EPOC) was analysed after a cycling protocol.

**Results:**

Mitochondrial capacity, as analysed using NIRS, was significantly higher in high-fitness individuals compared to low-fitness individuals in the gastrocnemius, but not in the FDS (*p* = 0.0036 and *p* = 0.20, respectively). Mitochondrial capacity in the gastrocnemius was significantly correlated with $\dot{V}$O_2_peak (*R*^2^ = 0.57, *p* = 0.0019). Whole body $\dot{V}$O_2_ recovery was significantly faster in the high-fitness individuals (*p* = 0.0048), and correlated significantly with mitochondrial capacity in the gastrocnemius (*R*^2^ = 0.34, *p* = 0.028).

**Conclusion:**

NIRS measurements can be used to assess differences in mitochondrial muscle oxygen consumption within a relatively normal, healthy population. Furthermore, mitochondrial capacity correlated with parameters of aerobic fitness ($\dot{V}$O_2_peak and EPOC), emphasising the physiological relevance of the NIRS measurements.

## Introduction

Muscle mitochondrial mass and function are positively affected by regular endurance exercise (Tonkonogi and Sahlin 2002). Due to the pivotal role of mitochondria in determining endurance capacity, there is a need for robust and non-invasive measurements of muscle mitochondrial function (Lanza and Nair 2009). Mitochondrial function in skeletal muscle is classically analysed ex vivo by measuring oxygen consumption in muscle biopsies. Less-invasive techniques have emerged over the last quarter century, allowing the measurement of mitochondrial function in vivo. These techniques are both based on the recovery of muscle homeostasis after exercise (Meyer 1988), assessed by measuring either the regeneration of phosphocreatine (PCr) using magnetic resonance spectroscopy (^31^P-MRS) or by the return of muscle oxygen consumption (m$\dot{V}$O_2_) to basal levels using near-infrared spectroscopy (NIRS). Mitochondrial function analysed by both techniques have been shown to be in good agreement with each other (Ryan et al. 2013), but NIRS offers advantages over ^31^P-MRS due to its higher portability and relatively low costs, making it more suitable for on-site and routine measurements.

NIRS uses the difference in light absorption of oxygenated and deoxygenated haemoglobin and myoglobin in the near-infrared region (Grassi and Quaresima 2016). By emitting light at different wavelengths, it is possible to differentiate between the oxygenated and deoxygenated states. When used on muscle and combined with arterial occlusions, it allows for measurement of m$\dot{V}$O_2_, as the change from oxygenated to deoxygenated haemoglobin and myoglobin reflects the use of oxygen in the tissue underneath the NIRS probe when blood flow is occluded (Van Beekvelt et al. 2001). Multiple, transient arterial occlusions after a short bout of exercise allows for the measurement of post-exercise recovery of m$\dot{V}$O_2_, a procedure used to assess mitochondrial capacity (Motobe et al. 2004). The underlying assumption is that post-exercise regeneration of readily available energy carriers (i.e. ATP and PCr) is directly linked to aerobic metabolism and, therefore, a higher mitochondrial capacity will be associated with a faster return of m$\dot{V}$O_2_ to the pre-exercise state (McMahon and Jenkins 2002). Indeed, the NIRS procedure to assess recovery kinetics of m$\dot{V}$O_2_ in vivo showed a strong correlation with maximal ADP-stimulated respiration of permeabilised muscle fibres in situ (Ryan et al. 2014).

On a whole body level, the regeneration of readily available energy carriers is assumed to contribute to the transient elevation of whole body oxygen consumption (m$\dot{V}$O_2_) above resting values in the immediate post-exercise period, also known as excess post-exercise oxygen consumption (EPOC). EPOC can be divided into a rapid and a prolonged phase, in which the mechanisms that contribute to the elevated m$\dot{V}$O_2_ are different (Gaesser and Brooks 1984). In particular, the rapid phase is defined to reflect the myofibrillar consumption of the readily available energy substrates in the beginning of exercise, such as PCr and ATP, as well as the replenishment of tissue and haemoprotein oxygen stores and lactate removal (Chance et al. 1992; Børsheim and Bahr 2003). In accordance with an important role for PCr regeneration in EPOC, Rossiter et al. showed that whole body $\dot{V}$O_2_ is related to muscle PCr kinetics in the recovery phase (Rossiter et al. 2002). As the latter may be related to skeletal muscle mitochondrial capacity, an inverse relationship between EPOC and NIRS assessment of m$\dot{V}$O_2_, reflecting skeletal muscle mitochondrial function, can be hypothesised (Kemp et al. 2015). However, it should be noted that despite clear effects on mitochondrial capacity (Lanza and Nair 2009), the effect of endurance training status on EPOC is controversial, most likely as a result of methodological difficulties (Børsheim and Bahr 2003). When comparing low- with high-endurance capacity subjects, no research design can control for relative exercise intensity, total work and exercise duration at the same time. Still, by controlling for relative intensity and exercise duration, different $\dot{V}$O_2_ recovery dynamics between trained and untrained subjects have been observed (Short and Sedlock 1997). Thus, when using such an approach, post-exercise whole body VO_2_ recovery dynamics may be a reflection of aerobic fitness, and be related skeletal muscle mitochondrial capacity.

NIRS has been used as a non-invasive measure for muscle mitochondrial function in various clinical populations such as COPD and cystic fibrosis patients. In general, NIRS studies indicate skeletal muscle mitochondrial dysfunction under these pathological conditions (Adami et al. 2017; Willingham and McCully 2017). On the other hand, endurance athletes, characterised by a high whole body peak oxygen uptake ($\dot{V}$O_2_peak), showed a faster post-exercise recovery of m$\dot{V}$O_2_ than fully sedentary subjects (Brizendine et al. 2013). Still, the difference in $\dot{V}$O_2_peak between the two groups was considerable (74 vs 34 ml.kg-1.min-1, respectively). It would be of interest to study whether this technique is also sensitive enough to detect differences within a more normally active, healthy population, as it is as yet unclear to what extent NIRS assessment of skeletal muscle mitochondrial capacity is related to other established measures of oxidative metabolism related to exercise, such as $\dot{V}$O_2_peak and EPOC in a normally active, healthy population. This information would further support the applicability and physiological relevance of NIRS assessment of mitochondrial capacity.

The aim of this study is to measure mitochondrial function using NIRS in a recreationally active, healthy population divided into relatively low- and relatively high-aerobic fitness groups and relate it to parameters of aerobic fitness. The recovery of m$\dot{V}$O_2_ in both the frequently activated gastrocnemius muscle and in the often undertrained forearm will be measured (Hamner et al. 2010). We hypothesised that the recovery of m$\dot{V}$O_2_ and whole body $\dot{V}$O_2_ recovery, i.e. EPOC, is faster in the relatively high-fitness group. Furthermore, we expect muscle and whole body $\dot{V}$O_2_ recoveries to correlate, since post-exercise replenishment of energy stores in the muscle encompasses an important component in the rapid phase of EPOC.

## Materials and methods

### Subjects

Healthy males between the age of 18 and 28 years were recruited from the local university and community population. None of the subjects had a history of cardiovascular, respiratory or metabolic disease. None of the subjects identified as regular smoker ( > 5 cigarettes per week) used recreational drugs during the study or reported recent use of performance enhancing drugs or supplements. Subjects were non-anaemic (haemoglobin concentration > 13 g/dL), verified using HemoCue Hb 201 microcuvette (HemoCue AB, Sweden). Main exercise modalities in high-fitness group were cycling (3x), lacrosse (2x), triathlon (1x), rowing (1x) and running (1x). Main exercise modalities in low-fitness group were sailing (1x), running (1x), weight lifting (1x), volleyball (1x) or no regular exercise at all. Only males were selected in this study due to the limited penetration depth of the NIRS device used and sex differences in subcutaneous adipose tissue thickness.

### Pre-experimental screening protocol

Subjects were selected based on whole body peak oxygen uptake ($\dot{V}$O_2_peak) measured using an incremental exercise test on electrically braked bicycle ergometer (Corival CPET, Lode, The Netherlands). Subjects were asked to refrain from vigorous exercise for 48 h and to have consumed their last meal 2 h before this test. Oxygen consumption, carbon dioxide production and air flow were measured using MAX-II metabolic cart (AEI technologies, USA). Exhaled air was continuously sampled from a mixing chamber and heart rate was measured with a strap-on chest heart rate monitor (Polar Electro, Finland). After 3 min of warming-up, the protocol started at a workload of 75 W, or 125 W for subjects who exercised > 3 times a week, and was increased every minute in increments of 25 W. Subjects were instructed to maintain a self-selected pedal rate between 70 and 80 revolutions per minute (RPM). Inability to pedal at a rate above 60 RPM for 15 s was considered point of exhaustion and the end of the test. For the test to be valid, two out of three of the following criteria should have been met: (1) A maximal heart rate within 10 beats of the predicted maximum (220—age), (2) attainment of a plateau in $\dot{V}$O_2_, i.e. $\dot{V}$O_2_ failing to increase with 150 mL/min, despite an increase in work load, (3) achievement of an RER ≥ 1.1. $\dot{V}$O_2_peak was determined by binning data in 15-s intervals.

Eight relatively high-aerobic fitness ($\dot{V}$O_2_peak ≥ 57 mL/kg/min) and eight low-aerobic fitness subjects ($\dot{V}$O_2_peak ≤ 47 mL/kg/min) were selected to take part in the study, based on chosen cut-offs. A total of 24 subjects were screened to end up with the desired sample size.

### Experimental protocol

All measurements were done fasted, i.e. subjects were not allowed to eat after 08:00 PM the night before. The subjects refrained from heavy physical exercise 48 h prior to testing and from any exercise and consumption of alcohol 24 h prior to testing. Maximal Voluntary Contraction (MVC) hand grip strength of the dominant hand was measured using a Jamar Hydraulic Hand Dynamometer (Performance Health, IL, USA). Highest value out of three 5-s isometric contractions was set as MVC. Body fat percentage was measured according to the four-site method by Durnin–Womersley using the skinfold measurements of the triceps, biceps, subscapula and suprailiac, measured using a skinfold caliper (Harpenden, UK). Furthermore, skinfold between NIRS receiver and transmitter was measured on the calf and the forearm.

### NIRS measurements

Deoxyhaemoglobin (HHb) and oxyhaemoglobin (O_2_Hb) were continuously measured using the continuous wave PortaMon wireless, dual-wavelength NIRS system (760 and 850 nm; PortaMon, Artinis Medical Systems, Netherlands). The 40-mm channel was used for analysis. Data were collected via bluetooth at 10 Hz using Oxysoft software (Artinis Medical Systems). The NIRS probe was placed longitudinally on the lateral gastrocnemius 4 cm distal to the knee joint and on the flexor digitorum superficialis (FDS). To secure the probe and protect it from environmental light, the probe was tightly taped to the skin. To measure oxygen consumption, a blood pressure cuff (Hokanson SC5 and SC12; D.E. Hokanson Inc., Bellevue, WA) was placed proximally of the probe above the knee joint and on the upper arm. The cuff was powered and controlled by a rapid cuff inflator system (Hokanson E20 and AG101 Air source; D.E. Hokanson Inc.) set to a pressure of 230–250 mm Hg. Post-exercise muscle oxygen consumption recovery was assessed similar to previously published protocols (Ryan et al. 2013). In summary, the protocol consists of three 30-s rest measurements of basal oxygen consumption. To calibrate the signal between individuals, the minimal oxygenation of the tissue underneath the probe was then determined by 30-s maximal hand grip exercise for FDS or by plantar flexion exercise using a rubber resistance band for gastrocnemius, followed by an arterial occlusion until baseline or with a maximum of 4 min total occlusion time. The hyperaemic response after the cuff was released was considered maximal oxygenation. Recovery oxygen consumption after exercise was measured after 30 s of intermittent handgrip exercise at 50% of MVC for the FDS or plantar flexion exercise using a rubber resistance band until 50% of maximal oxygenation for gastrocnemius. Right after exercise, a series of transient occlusions (5 × 5 s on/5 s off, 5 × 7 s on/7 s off, 10 × 10 s on/10 s off) was used to measure the recovery of muscle oxygen consumption after exercise. Recovery measurements were performed in duplicates with 2-min rest between tests.

### Analysis of muscle oxygen consumption data

NIRS data were analysed using Matlab-based (The Mathworks, MA, USA) analysis software (NIRS_UGA, GA, USA). Data were analysed as 100% of maximal oxygenation. m$\dot{V}$O_2_ was calculated during every arterial occlusion using the slope of the change in HHb and O_2_Hb (Hb difference) for 3 s for the 5-s occlusions, for 5 s for the 7-s occlusions, 7 s for the 10-s occlusions and 15 s for the basal measurements. A blood volume correction factor was used for each data point (Ryan et al. 2012) to correct for redistribution of blood distally from the cuff. In short, changes in HHb and O_2_Hb should be proportional during arterial occlusions. A blood volume correction factor (*β*) was calculated to account for possible changes and was used to correct each data point. m$\dot{V}$O_2_ recovery measurements post-exercise were fitted to a mono-exponential curve:

$$y~\left( t \right) = {\text{End}} - ~\Delta *e^{{ - k \cdot t}}$$

with *Y* representing the m$\dot{V}$O_2_ during the arterial occlusions, End being the m$\dot{V}$O_2_ immediately after the cessation of exercise, delta (∆) being the difference between m$\dot{V}$O_2_ after exercise and m$\dot{V}$O_2_ during rest, *K* being the rate constant expressed in time units, and *t* being time. Rate constants of duplicates were averaged. Rate constants calculated from curve fitting with *R*^2^ < 0.95 were excluded from analysis as a measure of poor data quality.

### EPOC measurements

Basal oxygen consumption (method see below) was measured in supine position after an overnight fast. Subject was rested 30 min before the facemask was attached. After 20 min of basal measurement, the subject cycled for 20 min at a work rate adjusted to 55% of $\dot{V}$O_2_peak (Maresh et al. 1992). This protocol resulted in equal relative intensity for each subject. Upon cessation of exercise, subjects were placed in supine position for 20 min.

### Analysis of whole body oxygen consumption data

Exhaled air was continuously sampled using a strap-on face mask, and binned in 15-s intervals. Due to the individualization of the exercise protocol, absolute oxygen consumption was different between subjects. The recovery of $\dot{V}$O_2_ expressed as a percentage of EPOC where the last 10 min of exercise was averaged and expressed as 100% EPOC and the values during the last 5 min of basal measurements were averaged and expressed as 0% EPOC (Short and Sedlock 1997). The recovery data were plotted using a two-phase exponential decay according to the formula:

$$Y = {\rm{Plateau}} + ({\rm{YOFast}}*\exp \left( { - K{\rm{Fast}}*X} \right) + ({\rm{YOslow}}*\exp \left( { - {\rm{KSlow}}*X} \right),$$

where

$${{\rm Y0}}{\text{Fast}} = \left( {{\rm Y0} - {\text{Plateau}}} \right)*{\text{PercentFast}}*0.01\;{\text{and}}$$

$${\rm YO}{\text{Slow}} = \left( {{\rm Y0} - {\text{Plateau}}} \right)*\left( {100 - {\text{PercentFast}}} \right)*0.01.$$

In the formula, Plateau represents the basal oxygen consumption, or 0% EPOC. Y0 represents the oxygen consumption during exercise, or 100% EPOC. KFast and KSlow represent the rate constants of recovery as an inverse unit of time. PercentFast is the fraction of the *Y* that is represented by the fast phase, as percent.

### Statistical analyses

Data are presented as mean ± SD. Statistical analyses were performed using GraphPad Prism v.5 (GraphPad Software, CA, USA). Means between the two groups were compared using a Students unpaired *t* test. Correlations between variables were compared using regression analysis. Significance was accepted at *p* < 0.05. Normality was tested using Shapiro–Wilk normality test. Not normal data were compared using Mann–Whitney tests.

## Results

All subjects completed all tests without any contraindications. Physical characteristics are shown in Table 1. All maximal exercise tests met at least two out of three criteria.

**Table 1:** Physical characteristics of the subjects

	Low fitness (*n* = 8)	High fitness (*n* = 8)
Age (years)	24.1 ± 2.7	22.6 ± 3.2
Weight (kg)	80.0 ± 8.3	73.3 ± 6.4
Height (m)	1.85 ± 0.05	1.79 ± 0.07
Fat mass (% of weight)	18.7 ± 2.8	12.5 ± 3.2**
$\dot{V}$O_2_ (mL/kg/min)	42.5 ± 3.9	62.5 ± 4.1****
Baecke PA score	8.0 ± 0.9	9.5 ± 0.7**
Hemoglobin (g/dL)	15.5 ± 1.1	15.3 ± 1.2
Skinfold forearm	7.2 ± 2.8	5.0 ± 1.6
Skinfold calf	15.0 ± 2.2	9.1 ± 4.1**

Values are means ± SD

^*^p < 0.05, **p < 0.01, ****p < 0.0001

### Recovery of m$\dot{V}$O_2_ in gastrocnemius and flexor digitorum superficialis

**Fig. 1:**
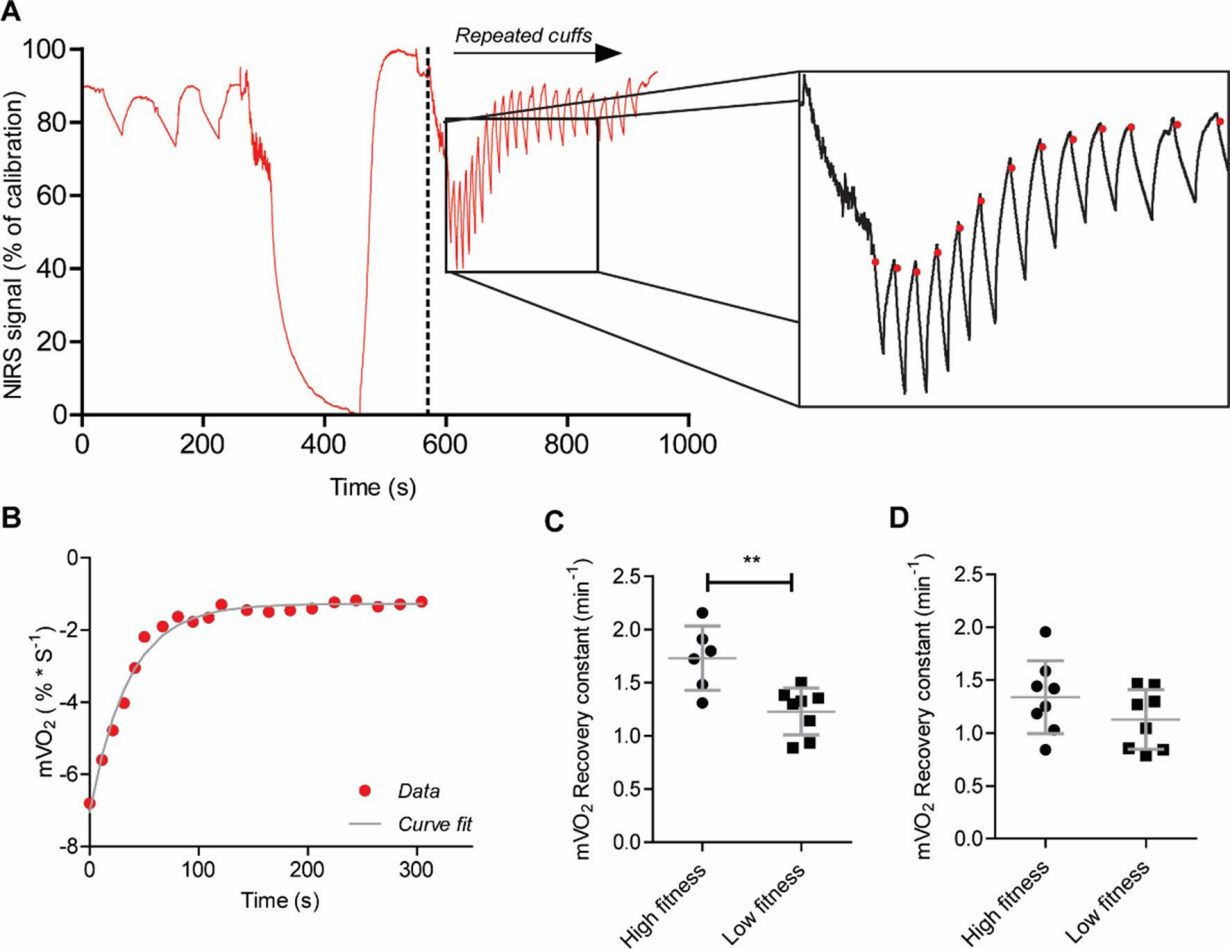
Representative plot of NIRS protocol. Red line represents NIRS signal of the Hb difference during protocol in percentage of maximal oxygenation, defined by a calibration procedure. Red dots represent the start of m$\dot{V}$O_2_ measurement for each occlusion (**a**) Curve fitting of m$\dot{V}$O_2_ recovery curve; red dots represent a single m$\dot{V}$O_2_ measurement. The grey line represents a monoexponential curve fit from which a recovery constant is derived (**b)**. m$\dot{V}$O_2_ recovery constants in high-fitness vs low-fitness groups. Recovery constants are derived from monoexponential curve fits of m$\dot{V}$O_2_ plots taking from NIRS measurements after 30 s of plantar flexion in exercise gastrocnemius **(c)** and 30 s of handgrip exercise in flexor digitorum superficialis **(d)**. Values are mean ± SD. **p < 0.005

**Fig. 2:**
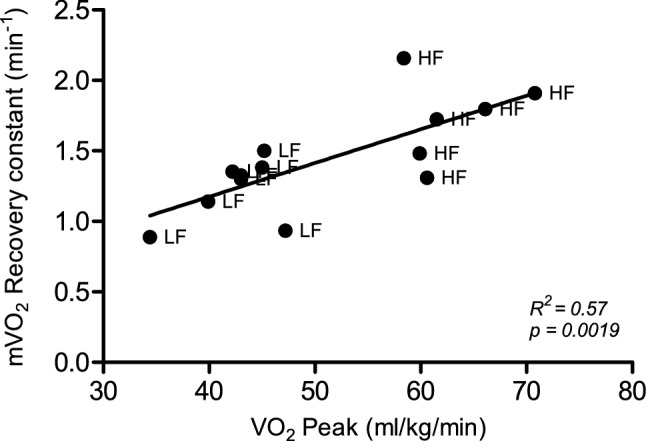
Correlation between recovery constants for muscle oxygen recovery in gastrocnemius calculated after 30 s of plantar flexion exercise using NIRS and maximal oxygen consumption ($\dot{V}$O_2_Peak) measured during an incremental exercise test

Recovery of oxygen consumption was measured using repeated occlusions after a short exercise protocol in FDS and gastrocnemius (Fig. 1a, b). Two NIRS data sets were excluded due to *r*^2^ < 0.95. Plateau for minimal oxygenation was reached in all individuals. Recovery rate constants were significantly different between high- and low-fitness groups for gastrocnemius (1.73 ± 0.30 vs. 1.23 ± 0.22, *p* = 0.0036; Fig. 1c), but not for the flexor digitorum superficialis (1.34 ± 0.35 vs. 1.13 ± 0.28, *p* = 0.20; Fig. 1d). The recovery constant of the gastrocnemius was significantly correlated with $\dot{V}$O_2_peak (Fig. 2; *R*^2^ = 0.57, *p* = 0.0019). In the FDS this correlation was not observed (*R*^2^ = 0.06, *p* = 0.32).

### Recovery of whole body $\dot{V}$O_2_ (EPOC)

**Fig. 3:**
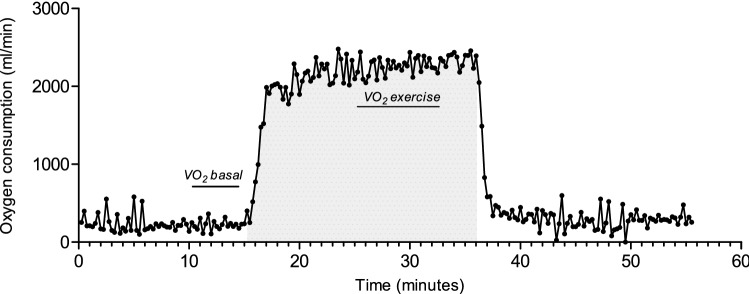
Representative measurement of oxygen consumption and recovery during the EPOC protocol. Oxygen consumption during rest and recovery was measured in supine position. Grey area reflects time spent cycling at 55% of $\dot{V}$O_2_peak. Oxygen consumption during basal state is indicated with ‘$\dot{V}$O_2_ basal’ and during exercise with ‘$\dot{V}$O_2_ exercise’

**Fig. 4:**
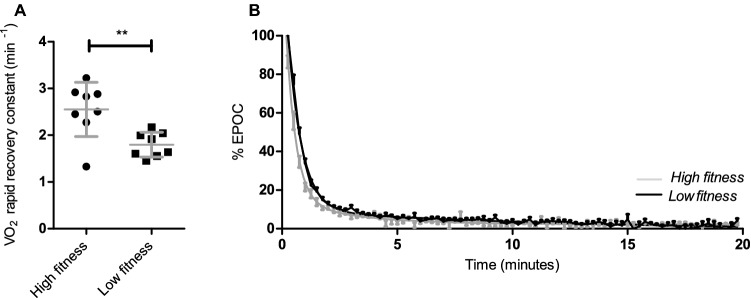
Recovery rate constants of the rapid recovery phase of EPOC fitted to two-phase exponential decay after 20-min cycling at 55% of $\dot{V}$O_2_peak (**a**). Recovery of EPOC presented as percentage of $\dot{V}$O_2_ during exercise as an average per group; high fitness (grey) and low fitness (dark) (**b**). Values are mean ± SD. **p < 0.005

The recovery of whole body $\dot{V}$O_2_ was measured after a short cycling protocol in supine position on bed (Fig. 3). Pre-test resting oxygen consumption was not significantly different between the high-fitness (240 ± 37 ml/min) and low-fitness groups (229 ± 37 ml/min, *p* = 0.53). Due to the use of an individualised exercise protocol, oxygen consumption during exercise was significantly larger in the high-fitness group (2409 ± 273 ml/min) compared to the low-fitness group (1710 ± 203 ml/min, *P* < 0.0001). The relative intensity (as a percentage to one’s $\dot{V}$O_2_peak was not different between the high-fitness (54 ± 7%) and the low-fitness groups (51 ± 6%; *p* = 0.47). EPOC volume was not significantly different between the high-fitness and low-fitness groups (576 ± 241.5 vs 2210 ± 268.8, *p* = 0.33). Fitting the recovery of $\dot{V}$O_2_ expressed as percentage of EPOC to a two-phase exponential decay resulted in a good fit (*R*^2^ = 0.95 ± 0.04). The rate constant of the rapid recovery phase was significantly different between the high- and low-fitness groups (Fig. 4; *p* = 0.0048), but not for the rate constant of the prolonged phase (0.34 ± 0.16 min ^−1^ vs. 0.18 ± 0.21, *p* = 0.13). The rate constant of the rapid recovery phase was significantly correlated with $\dot{V}$O_2_peak (*R*^2^ = 0.66, *p* = 0.0004).

### Relationship between m$\dot{V}$O_2_ and whole body $\dot{V}$O_2_ recovery

**Fig. 5:**
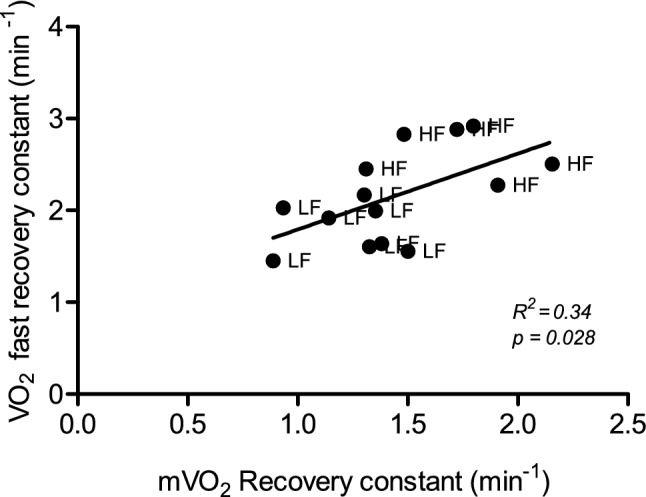
Correlation between rate constants of the rapid phase of recovery of whole body oxygen consumption after 20-min cycling protocol at 55% of $\dot{V}$O_2_peak and the recovery of muscle oxygen consumption of the gastrocnemius after 30-s low-intensity plantar flexion exercise measured using NIRS. *HF* High fitness, *LF* Low fitness

To test whether there is a relationship between the recovery of oxygen consumption of the muscle after a short exercise and that of the whole body after an exercise protocol, both conditions were analysed for correlation. Indeed, the recovery constant of gastrocnemius was significantly correlated with the recovery constant of the rapid phase whole body m$\dot{V}$O_2_ (Fig. 5; *R*^2^ = 0.34, *p* = 0.028).

## Discussion

Our results show that the present NIRS protocol was able to discriminate high-fitness and low-fitness individuals within a relatively homogenous group, i.e. young, healthy and normally active males, based on post-exercise m$\dot{V}$O_2_ recovery. Recovery during the rapid phase of the EPOC was faster in the high-fitness group compared to the low-fitness group. Furthermore, a correlation between whole body oxygen recovery and m$\dot{V}$O_2_ recovery was found in the gastrocnemius muscle, pointing out the physiological relevance for NIRS assessment of m$\dot{V}$O_2_ recovery.

### Relationship between m$\dot{V}$O_2_ recovery and aerobic fitness

In the high-fitness group, recovery of oxygen consumption in gastrocnemius muscle was faster than in the low-fitness group indicating that mitochondrial capacity of that specific muscle was significantly higher in the high-fitness group compared to the low-fitness group. Our findings are in line with previous assessment of mitochondrial capacity measured using NIRS in highly trained endurance athletes and inactive individuals, showing an approximate doubling of mitochondrial capacity in the athlete group (Brizendine et al. 2013). However, with an average $\dot{V}$O_2_ peak of 34 and 74 mL Kg^−1^ min^−1^ for the inactive subjects and endurance athletes, respectively, the difference in endurance capacity, and thus aerobic adaptations, was substantially larger than in the current study. Thus, the present NIRS protocol showed adequate sensitivity to detect the (considerably smaller) difference in mitochondrial capacity between the high- and low-fitness individuals within our relatively homogenous population of healthy, normally active, young males. Our results, therefore, further widen the application of this non-invasive technique for in vivo human metabolic research.

Our results also correlate well with ex vivo measurements of skeletal muscle mitochondrial capacity in trained and untrained male subjects, with an average age and $\dot{V}$O_2_ peak remarkably similar to our study (Phielix et al. 2012). Maximal mitochondrial respiration of permeabilised vastus lateralis muscle fibres from trained subjects was approximately 50% higher when compared to untrained subjects. Endurance training increases aerobic capacity due to, among other mechanisms, an increase in mitochondrial mass and more regular exposure to local exercise stimuli most likely induced mitochondrial biogenesis and increased mitochondrial capacity in the high-fitness group (Ljubicic et al. 2010). This increased mitochondrial capacity may be physiologically relevant since, when taking both groups together, mitochondrial capacity of the gastrocnemius was significantly correlated with $\dot{V}$O_2_Peak. Our results are in agreement with a study in elderly subjects showing a comparable correlation between ex vivo mitochondrial oxidative capacity of permeabilised muscle fibres obtained from a vastus lateralis biopsy and $\dot{V}$O_2_ peak (Coen et al. 2013). Still, as ageing is generally associated with a decline both in $\dot{V}$O_2_ peak and mitochondrial capacity (Conley et al. 2000), this latter study does not necessarily reflect a correlation between endurance training status and skeletal muscle mitochondrial capacity in young adults. A ^31^P-MRS study in adult, but not elderly, women revealed a significant correlation between the rate of PCr resynthesis and $\dot{V}$O_2_ max (Larson-Meyer et al. 2000), and in young males cytochrome C oxidase activity of vastus lateralis muscle tissue correlated significantly with $\dot{V}$O_2_ max (Booth and Narahara 1974). Hence, the results of our NIRS study, showing a relatively strong correlation between in vivo oxidative capacity of the muscle and $\dot{V}$O_2_ peak, are in line with the aforementioned studies. Therefore, the current study further establishes the application of NIRS to assess skeletal muscle mitochondrial capacity in vivo, and provides novel additional data supporting the physiological relevance of skeletal muscle mitochondrial capacity for aerobic performance.

### Relationship between gastrocnemius m$\dot{V}$O_2_ recovery and whole body $\dot{V}$O_2_ recovery

A faster rapid whole body oxygen recovery after 20 min of cycling at 55% of $\dot{V}$O_2_Peak was shown to be correlated with a faster m$\dot{V}$O_2_ recovery in the gastrocnemius. Although various adaptations to endurance training could underlie the significant difference of the rapid recovery phase of the whole body $\dot{V}$O_2_ recovery between the two groups, such as enhanced blood flow and increased vascularization of the muscle (Egan and Zierath 2013), the significant correlation suggests a role for mitochondrial capacity in post-exercise $\dot{V}$O_2_ recovery. Both NIRS and EPOC, at least in part, reflect the ability of the mitochondria to replenish readily available energy substrates post-exercise using oxidative metabolism. It should be noted, however, that the correlation between post-exercise recovery of whole body oxygen and skeletal muscle mitochondrial capacity as assessed by NIRS was less strong than the correlation between $\dot{V}$O_2_peak and mitochondrial capacity, as became apparent from the lower *R*^2^ values. In other words, the contribution of skeletal muscle mitochondrial capacity to the post-exercise $\dot{V}$O_2_ recovery dynamics of the current protocol is smaller than its contribution to $\dot{V}$O_2_peak, suggesting that other mechanisms, such as lactate removal, also play an important role in post-exercise $\dot{V}$O_2_ recovery. The ability of NIRS to discriminate between two groups with distinct aerobic fitness levels and the correlation with an established recovery parameter supports the use of measuring m$\dot{V}$O_2_ recovery using NIRS as a relevant physiological parameter to reflect mitochondrial capacity.

To assess the effect of increased mitochondrial capacity on recovery of whole body $\dot{V}$O_2_ recovery, a short and moderate-intensity exercise protocol was used. Short and moderate-exercise protocols have been shown to induce a rapid EPOC component, while limiting an increase in plasma lactate concentration and body temperature (Maresh et al. 1992). Accordingly, while cycling at the same relative intensity, 55% of their $\dot{V}$O_2_peak for a duration of 20 min, absolute oxygen consumption was higher in high-fitness individuals due to their higher $\dot{V}$O_2_peak and higher fat free mass. Possibly, the initial $\dot{V}$O_2_ at the end of exercise and the start of the EPOC could affect the rapid phase of recovery. To correct for this, oxygen consumption during exercise was set to 100% and the EPOC expressed as percentage of recovery to baseline values. Alternatively, one could opt for an absolute intensity protocol, in which the magnitude of the oxygen debt at the end of the exercise protocol is equal, regardless of endurance capacity. Yet, the duration of exercise to reach a specific absolute oxygen debt would not be comparable and EPOC is also known to be affected by exercise duration and intensity; this has been extensively reviewed elsewhere (Børsheim and Bahr 2003).

The difference in post-exercise whole body $\dot{V}$O_2_ recovery between the high-fitness and low-fitness groups, as observed in the current protocol controlled for relative exercise intensity, is in agreement with a previous study of Short et al., who reported a difference in fast recovery of $\dot{V}$O_2_ between trained and untrained subjects when EPOC is expressed as a percentage of recovery to baseline (Short and Sedlock 1997). Likewise, a faster EPOC recovery was reported in trained women compared to untrained women after 300-kcal cycle test at 65% of $\dot{V}$O_2_peak (Frey et al. 1993). On the other hand, several studies using an absolute EPOC protocol found no differences in EPOC in trained and untrained individuals (Brehm and Gutin 1986; Sedlock 1994). Due to these different study outcomes, probably resulting from the different experimental setups, there is no clear consensus on the effects of (endurance) training status on EPOC. However, in agreement with the above-mentioned results, our results, not only show a faster $\dot{V}$O_2_ recovery in high-fitness group but also show a significant correlation between skeletal muscle mitochondrial capacity and $\dot{V}$O_2_ recovery, provide novel supporting evidence that post-exercise $\dot{V}$O_2_ recovery using a protocol controlling for relative exercise intensity, may reflect aerobic fitness. This notion is further substantiated by the significant correlation between this parameter and $\dot{V}$O_2_peak in the present study.

### m$\dot{V}$O_2_ recovery in FDS and gastrocnemius

Besides local effects of exercise, one-legged cycling exercise can also trigger responses in muscles that are not engaged in exercise, as was shown by Catoire et al. (Catoire et al. 2012). We hypothesised that high-fitness individuals could, therefore, also have increased mitochondrial capacity in muscle groups that are not directly activated by exercise, such as the FDS muscle in the forearm, but are indirectly activated through systemic effects of exercise. Yet, although mitochondrial capacity was higher in the gastrocnemius of high-fitness individuals compared to low-fitness individuals, the difference in mitochondrial capacity in FDS was smaller but not significant with a *p* value of 0.2. It could be that the effects of systemic exercise on the indirectly stimulated muscle is too small to be detected by the current sample size, or that those systemic effects on mitochondrial capacity did not occur.

In a previous study, mitochondrial capacity was analysed in the vastus lateralis muscle (Brizendine et al. 2013). The current study is the first to analyse differences in mitochondrial capacity comparing endurance capacity using the gastrocnemius instead. Measurements in the vastus lateralis are done with a cuff placed high up on the leg which is generally considered more uncomfortable for a subject than measurements in the gastrocnemius, where the cuff is placed just below or above the knee joint. This measurement technique likely increases the throughput and tolerability of the NIRS measurement, which will ensure reliability of the data and possibly lowers variability between measurements.

## Conclusion

This study provides evidence that NIRS measurements can be used to assess differences in mitochondrial muscle oxygen consumption within a relatively normal, healthy population. In a normally active population, mitochondrial capacity was significantly higher in high-fitness individuals with a relatively high $\dot{V}$O_2_peak. Furthermore, mitochondrial capacity correlated with $\dot{V}$O_2_peak, but also to post-exercise whole body $\dot{V}$O2 recovery, emphasising the physiological relevance of the NIRS measurements. The observed correlation between skeletal muscle mitochondrial capacity and whole body $\dot{V}$O_2_ recovery supports the delineation of this latter measure as a parameter of aerobic fitness. Future research that aims to study mitochondrial capacity could use the non-invasive nature of NIRS, its relative affordability and increased portability, which allows it to be easily applied to assess mitochondrial functionality to study effects of life style and/or dietary interventions.

## Abbreviations

EPOC: Excess post-exercise oxygen consumption
FDS: Flexor digitorum superficialis
HHb: Deoxyhaemoglobin
mVO_2_: Muscle oxygen consumption
NIRS: Near-infrared spectroscopy
O_2_Hb: Oxyhaemoglobin
^31^P-MRS: Magnetic resonance spectroscopy
RPM: Revolutions per minute
$\dot{V}$O_2_max: Maximal oxidative capacity
$\dot{V}$O_2_peak: Whole body peak oxygen uptake
